# Short-Time High-Oxygen Pre-Treatment Delays Lignification of Loquat (*Eriobotrya japonica* Lindl.) During Low-Temperature Storage

**DOI:** 10.3390/foods14020201

**Published:** 2025-01-10

**Authors:** Runlei Kou, Mengfei Peng, Jiaxuan Zheng, Shuangdi Hou, Linyuan Ma, Xia Liu

**Affiliations:** 1 State Key Laboratory of Food Nutrition and Safety, Key Laboratory of Food Nutrition and Safety, Ministry of Education of China, College of Food Science and Engineering, Tianjin University of Science & Technology, Tianjin 300457, China; m13341289195@163.com (R.K.); pengmengfei99@163.com (M.P.); zhengjia_xuan@126.com (J.Z.); mly2000221@163.com (L.M.); 2 Key Laboratory of Agricultural Products Storage and Preservation, Ministry of Agriculture and Rural Affairs, Research Institute of Agricultural Products Preservation and Processing, Tianjin Academy of Agricultural Sciences, Tianjin 300384, China; houshuangdi321@163.com

**Keywords:** loquat, short-time high-oxygen pre-treatment (SHOP), lignification, lignin synthesis enzyme, fruit quality

## Abstract

Lignification often occurs during low-temperature storage in loquat fruit, leading to increased firmness and lignin content, water loss, and changes in flavor. As induced stress factors, short-time high-oxygen pre-treatment (SHOP) can initiate resistant metabolism and regulate the physicochemical qualities during fresh fruit storage. However, the effect of SHOP on the lignification and quality of loquat has been reported less. In the present study, loquat fruit was immersed in oxygen concentrations of 70%, 80%, and 90% for 30 min before being stored at 4 ± 1 °C. The results showed that the 80% SHOP samples had lower lignin accumulation and firmness, showing reductions of 23.1% and 21.1% compared to the control at 50 days. These effects were associated with the inhibition of the activities of lignin synthesis-related enzymes, including phenylalanine ammonia-lyase (PAL), 4-coumarate-CoA ligase (4CL), cinnamyl alcohol dehydrogenase (CAD), and peroxidase (POD). Meanwhile, 80% SHOP improved the antioxidant enzyme system and maintained the structural integrity of the cells. Furthermore, SHOP retained the color and suppressed decay and weight loss and the decline in the soluble solids content (SSC) and titratable acidity (TA). As a convenient and cheap physical approach, SHOP is a promising technology for delaying lignification by regulating lignin synthesis in loquat storage.

## 1. Introduction

Loquat (*Eriobotrya japonica* Lindl.) is a non-climacteric fruit prized for its rich nutritional content, including minerals and carotenoids, as well as its medicinal properties, such as anti-inflammatory, antioxidant, and cough-relieving effects, making it a fruit of significant economic value [1,2]. Loquat is susceptible to decay after harvest and has a concise shelf life, leading to a short postharvest storage life of approximately 10 days when stored at room temperature and 30 days at low temperature [3,4]. Low-temperature storage is an effective method to extend the shelf life of fruit. Nevertheless, lignification often occurs at storage temperatures below 5 °C and seriously affects loquat quality and commercial value [5].

Lignification is widely recognized as a response to abiotic stress and natural senescence, primarily resulting from the accumulation of lignin [6]. It consists of polyphenolic polymers formed by the oxidative polymerization of monolignols (p-coumaryl alcohol, coniferyl alcohol, and sinapyl alcohol) through a pathway of phenylalanine metabolism [7]. Lignin synthesis involves phenylalanine ammonia lyase, Phenylalanine ammonia-lyase (PAL), 4-coumarate-CoA ligase (4CL), cinnamyl alcohol dehydrogenase (CAD), and peroxidase (POD) [7,8]. Furthermore, many reports revealed that the activities of PAL, 4CL, CAD, and POD, as well as the expression of these respective genes, affect lignin biosynthesis and are essential for the regulation of horticultural crop lignification, such as in kiwifruit, bamboo shoot, and water bamboo [9,10,11]. The above findings demonstrate that regulating PAL, 4CL, CAD, and POD activities is essential to delay loquat lignification.

The excessive accumulation of reactive oxygen species (ROS) leads to fruit deterioration [12]. There is a positive correlation between lignin deposition and reactive oxygen species (ROS) levels, suggesting that it plays a regulatory role in lignin biosynthesis [13,14,15,16]. Therefore, the antioxidant system, which plays an important role in preventing the excessive accumulation of ROS and mitigating oxidative damage, both senescence and fruit ripening, includes enzymes such as catalase (CAT), ascorbate peroxidase (APX), and superoxide dismutase (SOD) [17].

Various treatments, including 1-methylcyclopropene (1-MCP), melatonin, hot air, and chitosan coating, have been applied to inhibit lignification occurrence and preserve quality in loquat [18,19,20,21]. However, these methods are expensive, time-consuming, and not widely adopted in commercial storage. Oxygen treatment is a green, cheap, and convenient emerging technology. Oxygen is a signal of abiotic stress in response to activating plant defense mechanisms [22,23]. High-oxygen-modified atmosphere packaging (High-O_2_-MAP) has been proven to help regulate the lignification metabolism, decreasing fruit decay, and maintaining quality, while it is not appropriate for long-term storage since the prolonged use of high oxygen could cause severe damage to fresh produce tissues [24,25,26]. Therefore, it is hoped that a technology that takes advantage of high oxygen will be developed without side effects.

Short-time high-oxygen pre-treatment (SHOP) is a technology that has highlighted the potential to alleviate the deterioration of postharvest fruits and vegetables due to their advantages of avoiding oxygen damage and being simple to control [22,23]. In particular, the accumulation of phenylpropane metabolic pathways and reactive oxygen species (ROS) is related to lignin synthesis. Short-time high-oxygen pre-treatment has been shown to regulate the phenylpropane metabolic pathway and effectively reduce reactive oxygen species (ROS) accumulation [22,27]. Therefore, SHOP is considered a suitable technology for alleviating lignification.

As an utterly innocuous molecule in its ground state, SHOP is involved in various plant metabolic reactions. However, there is no available information about the effect of SHOP on lignification during low-temperature storage in loquat. In this study, the effectiveness of SHOP to delay lignification was evaluated by analyzing the lignin content, activities of key lignin synthesis enzymes, microstructure, and postharvest quality during loquat cold storage, which lays a theoretical foundation for the mechanism of SHOP and provides a new technology for loquat.

## 2. Materials and Methods

### 2.1. Materials and Treatment

**Figure 1:**
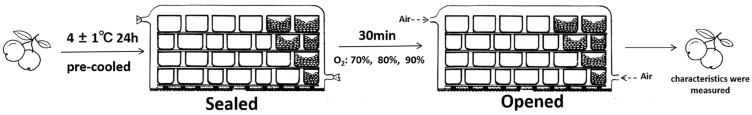
The experimental treatment processes of loquat.

The fruits of Loquat (*Eriobotrya japonica* L. cv. ‘Dawuxing’) were hand-picked at commercial maturity, which corresponded to the fourth stage of ripeness (orange) from an orchard in Qiannan, Guizhou Province, China [28,29,30]. After 24 h of pre-cooling at 4 ± 1 °C to rapidly remove field heat in harvested loquat to preserve their quality, the fruit was selected for consistent color and size without mechanical damage. Loquat fruits were randomly distributed into four groups, each with 600 loquats and three replications. As shown in Figure 1, loquats were first pre-cooled at 4 ± 1 °C for 24 h, then placed in sealed tents with 0.2 mm Polyethylene (PE). Three SHOP groups and the control group, each with three replications, were applied by injecting 70% oxygen (group 1), 80% oxygen (group 2), 90% oxygen (group 3), and normal air (group 4) into the sealed tents for 30 min. Afterward, the air inlet and outlet were opened, and the oxygen was replaced with normal air. Gas concentrations were monitored using an O_2_/CO_2_ analyzer (PBI-Dansensor A/S, Ringsted, Denmark), which provided data for the gas control system regarding when to terminate the O_2_ treatment based on the target concentration.

The oxygen treatments were applied a single time. Finally, loquat fruits were stored in tents with normal air at 4 ± 1 °C with 90 ± 2% relative humidity for 50 days. Fruits were chosen at random every 10 days for analysis and research.

### 2.2. Scanning Electron Microscopy (SEM)

The microstructure of the loquat was observed following the method described by Hou et al., with some modifications [22]. The microstructure of the loquat was observed by scanning electron microscopy (SU1510, Hitachi, Tokyo, Japan). Briefly, the flesh was chopped into 3 × 3 × 2 mm slices with a razor blade, which were refrigerated using liquid nitrogen and placed in a −80 °C refrigerator. Frozen samples were vacuum freeze-dried at −65 °C for 24 h. Finally, samples were spray-coated with gold (IB-3, EIKO, Tokyo, Japan) and observations were carried out at 250× magnification.

### 2.3. Firmness and Lignin Content

Firmness was measured with a digitized fruit firmness tester (GY-4, Toppu Instrument, Hangzhou, China) using a 6 mm probe diameter along the equatorial part. The firmness of the loquat was represented by the compressive force, which refers to the external force applied to the fruit during the measurement process. The results were given in Newtons (N). Lignin content was measured using the revised methodology [31]. After being homogenized in 4 mL of 95% (*v*/*v*) ethanol, 4 g of loquat sample was centrifuged at 10,000× *g* for 20 min. The precipitate was washed three times with 95% ethanol and n-hexane solutions (1:2, *v*/*v*) separately, then dried at 70 °C and resolved in 2 mL of glacial acetic acid with 25% acetyl bromide. The reaction was determined by adding 1 mL of 2 mol L^−1^ NaOH, 1 mL of glacial acetic acid, and 0.1 mL of 7.5 mol L^−1^ hydroxylamine hydrochloride. The absorbance at 280 nm was determined by a UV spectrophotometer (T6-1650E, Persee, Beijing, China), and data were presented using a fresh weight basis. Lignin content was expressed as ×10^3^ A_280_ kg^−1^, corresponding to the absorbance data.

### 2.4. Enzyme Activity

The PAL activity of loquat was assessed using a modified method [32]. Loquats (5 g) were added with 5 mL of extraction buffer (40 g L^−1^ PVPP, 2 mmol L^−1^ EDTA, 5 mmol L^−1^ mercaptoethanol) and ground into homogenate at low temperature, and then centrifuged at 10,000 r/min at 4 °C for 30 min to obtain the crude enzyme solution. Volumes of 3 mL of boric acid buffer (50 mmol L^−1^, pH 8.8) and 0.5 mL of L-Phenylalanine (20 mmol L^−1^) were kept warm at 37 °C for 10 min, and then mixed with 0.5 mL of enzyme solution. The absorbance value at 290 nm was measured before and after 60 min of heat preservation. The difference in absorbance was used to calculate PAL activity. This was repeated three times. PAL activity was expressed as U g^−1^ fresh weight (FW).

The POD activity in loquat was measured with slight modifications to a previous method [33]. Samples (5 g) were homogenized with 5 mL extraction buffer (1 mmol L^−1^ PEG, 4% PVPP, 1% Triton X-100) at low temperature, then centrifuged at 10,000 r/min for 30 min at 4 °C to obtain the enzyme solution. Volumes of 3 mL of guaiacol (25 mmol L^−1^) and 0.5 mL enzyme solution were added, followed by 200 µL of H_2_O_2_ (0.5 mol L^−1^) for the reaction. The absorbance value at 470 nm was measured every 1 min six consecutive times. The POD activity was calculated based on the change in absorbance value. The experiment was repeated three times. POD activity was expressed as U g^−1^ fresh weight (FW).

The 4CL activity was measured following the method reported by Li et al. [34]. Loquat samples (0.7 g) were ground with 3.0 mL of 4CL extraction buffer (0.1 mmol L^−1^ DTT, 25% glycerol, pH 8.0) to a homogeneous paste. The homogenate was centrifuged at 10,000× *g* for 30 min at 4 °C, and the supernatant was as the sample extract. Volumes of 0.45 mL of MgCl_2_ (75 mmol L^−1^), 0.15 mL of adenosine triphosphate (50 mmol L^−1^), 0.15 mL of p-Coumaric acid (5 mmol L^−1^), 0.15 mL of coenzyme A (1 mmol L^−1^), and 0.5 mL of the sample extract were mixed and incubated at 40 °C for 60 min. The reaction was stopped with 100 μL of 6 mol L^−1^ HCl, and absorbance was measured at 333 nm. Distilled water was used as the blank. Each sample was measured in triplicate. 4CL activity was expressed as U g^−1^ fresh weight (FW).

CAD activity was quantified, as described by Cai et al., with specific changes [18]. A volume of 3 mL of phosphate buffer (100 mmol L^−1^, pH 6.5) was taken to homogenize 1 g of the loquat sample, then centrifuged at 10,000× *g* for 30 min. The reaction mixture containing 0.2 mL of enzyme extract, 1.8 mL of phosphate buffer (100 mmol L^−1^, pH 6.5), 1.0 mL of NADP (8 mmol L^−1^), and 1.0 mL of trans-cinnamic acid (4 mmol L^−1^) was in incubation for 30 min at 37 °C. The reaction was stopped with 0.1 mL hydrochloric acid (6 N) HCl, and absorbance at 340 nm was assayed. Enzyme activities were presented in U g^−1^ of the fresh sample weight.

### 2.5. Reactive Oxygen Species (ROS)-Related Enzyme Activity and MDA Content

The activities of superoxide dismutase (SOD) and catalase (CAT) in loquat were determined using the method described by Zhang et al. and slightly modified [35]. A 5 g sample of loquat was homogenized in 5 mL of extraction buffer (5 mmol L^−1^ DTT, 5% PVP). The homogenate was centrifuged at 12,000× *g* for 30 min at 4 °C, and the supernatant was collected. Then, 1.7 mL of 50 mmol L^−1^ sodium phosphate buffer (pH 7.8), 0.3 mL of 130 mmol L^−1^ MET solution, 0.3 mL of 0.75 mmol L^−1^ NBT solution, 0.3 mL of 0.1 mmol L^−1^ EDTA-Na_2_ solution, and 0.3 mL of 0.02 mmol L^−1^ riboflavin solution were mixed and reacted with 50 μL of the enzyme extract. The absorbance of the reaction mixture at 560 nm was measured after subjecting the tubes to light for 15 min, with one test tube kept in the dark as a blank control. The SOD activity in the samples was expressed as U g^−1^.

The same enzyme extract with SOD was used to determine the activities of CAT. In a 10 mL test tube, 100 μL of the supernatant was sequentially added to 2.9 mL of 20 mmol L^−1^ H_2_O_2_ for the reaction. The absorbance was measured at 240 nm. The CAT activity in the samples was expressed as U g^−1^.

The method was modified based on Yang’s approach for determining ascorbate peroxidase (APX) activity [36]. A 5 g sample of loquat was homogenized in 5 mL of extraction buffer (0.1 mmol L^−1^ EDTA, 1 mmol L^−1^ ascorbic acid, and 2% PVPP), then centrifuged at 12,000× *g* for 30 min at 4 °C to collect the supernatant. The reaction mixture consisted of 2.6 mL of reaction buffer, 0.3 mL of 30% H_2_O_2_, and 100 μL of the enzyme extract. The absorbance was measured at 290 nm. The APX activity in the samples was expressed as U g^−1^.

MDA content was assayed based on the reported approach of Qiao et al. [33]. A mass of 1.0 g of loquat fruit was ground with 5.0 mL of trichloroacetic acid solution (100 g L^−1^) until a homogeneous slurry was obtained, which was then centrifuged at 10,000× *g* for 20 min at 4 °C to collect the supernatant. Next, 2.0 mL of the sample extract was added to a centrifuge tube, followed by 2.0 mL of thiobarbituric acid solution (0.6%). Trichloroacetic acid solution served as the blank. The mixture was shaken and heated in a boiling water bath for 20 min, then cooled. After centrifuging at 10,000× *g* for 20 min at 4 °C, absorbance was measured at 450, 532, and 600 nm. The procedure was repeated three times. The result was presented in mmol kg^−1^ of fresh weight.

### 2.6. Color Properties

The surface color of loquat fruit, including lightness values (L*), red-green values (a*), and yellow-blue values (b*), was determined using a chromameter (CM-3600d, Konica Minolta, Tokyo, Japan). ΔE* was evaluated through a comparison to the initial fruit values to assess the color change of the fruit: $\Delta E*=\sqrt{{\Delta L*}^{2}+{\Delta a*}^{2}+{\Delta b*}^{2}}$ [37].

### 2.7. Decay Rate and Weight Loss

Fruit decay was evaluated by the presence of brown spots of soybean grain size and a change in the skin color from yellow to brown. The decay rate was computed using the following formula: $N_{1}/N_{2}\;\times \;100\%$. Here, N_1_ represents the number of decayed samples, and N_2_ indicates the overall number of samples measured. Weight loss was accounted for using the following equation: $(W_{1}\;-\;W_{2})/W_{1}\;\times \;100\%$. Here, W_1_ denotes the weight of fruit pre-storage, and W_2_ shows the weight of fruit post-storage.

### 2.8. Soluble Solids Content (SSC) and Titratable Acidity (TA)

SSC was assayed with a pocket refractometer (PAL-1, ATAGO, Tokyo, Japan). The result was expressed as a percentage. TA was assayed using acid–base titration [38]. A volume of 20 mL of juice was titrated with 50 mmol L^−1^ NaOH solution to pH 8.2. The result was shown as malic acid as a percentage.

### 2.9. Statistical Analysis

Each experiment treatment was performed in triplicate. One-way analysis of variance (ANOVA) was conducted using SPSS 26.0. Data were presented as mean ± SD and analyzed for significance using the LSD test. *p* ≤ 0.05 was considered significant.

## 3. Results and Discussion

### 3.1. Effect of SHOP on Appearance and SEM Observation

**Figure 2:**
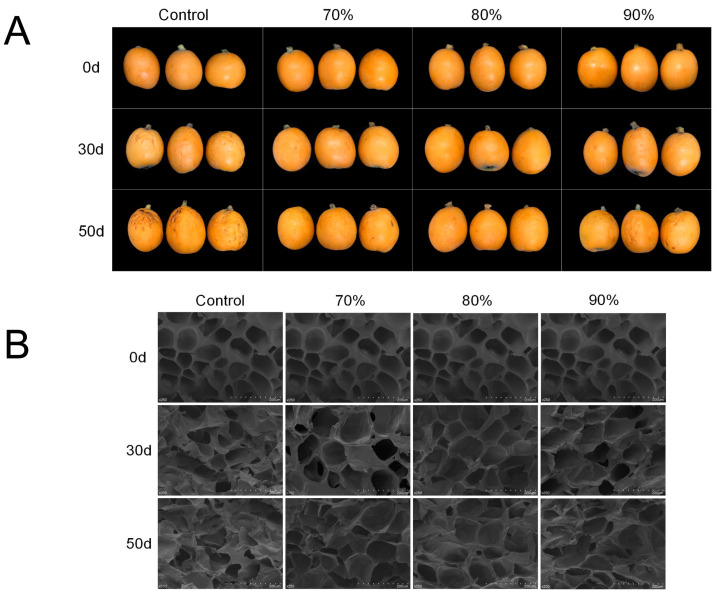
Effect of short-time high-oxygen pre-treatment (SHOP) on the appearance (**A**) and morphology (**B**) of loquat fruit. The microstructure of loquat was observed with scanning electron microscopy at 250× magnification.

The appearance of loquat fruit with different SHOP treatments during storage from 0 to 50 d is shown in Figure 2A. This indicated that SHOP could inhibit the surface water loss of loquat. Notably, the 80% SHOP fruit had the smoothest appearance and retained their color well. In contrast, color loss and browning on storage day 30 and water loss, shrinkage, and partial decay symptoms on day 50 were observed on the control fruit.

Cell wrinkles were observed in SEM images related to cell wall degradation and the increase in lignified cells in the plant tissues [39]. As shown in Figure 2B, changes in the microstructure of the central loquat pulp were observed by SEM. At the beginning of storage, the flesh cell had a compact structure and regular shape, and the cell walls were intact. After 30 days of storage, the cell walls were swollen and separated, and the angularity of the cell outlines progressively vanished. The control fruit was severely impaired in terms of the flesh tissue. In the meantime, the structure appeared to fold, deform, and collapse after 50 days of storage. On the contrary, SHOP exerted a function in preserving the integrity and uniformity of the loquat tissue structure.

In the present study, the cell walls gradually thickened, and apparent cell rupture occurred with increased storage time [22,39]. Compared to the control, the SHOP treatment more effectively reduced cell damage. Notably, the 80% SHOP fruit showed a better microstructure than the 90% SHOP fruit. Lignin accumulation thickens the cell walls and affects the microstructure of the cells [39]. Furthermore, the high-oxygen damage caused by the 90% SHOP treatment may trigger a stress response, resulting in an imbalance of the membrane permeability and membrane damage, which can lead to the collapse of the cell structure [26]. The optimal oxygen concentration of 80% may reduce lignification accumulation and cell damage to preserve the microstructural integrity.

### 3.2. Effect of SHOP on Firmness and Lignin Content

**Figure 3:**
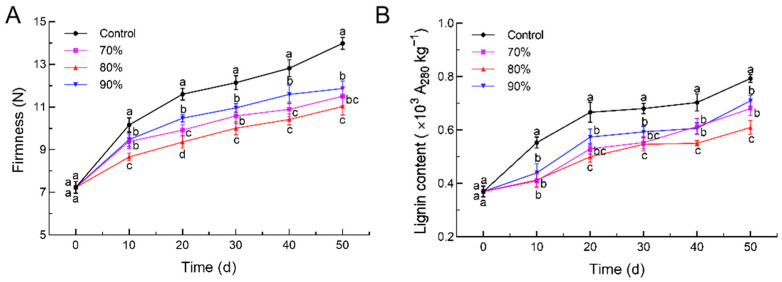
Effect of short-time high-oxygen pre-treatment (SHOP) on firmness (**A**) and lignin content (**B**) of loquat fruit. Data are expressed as mean ± standard deviation. Different letters on each sampling day denote significant differences among four groups (*p* ≤ 0.05).

The firmness of loquat fruit progressively increases during storage, a phenomenon thought to be attributed to tissue lignification [40]. The firmness and lignin content of the loquat were gradually increased during the 50 days of storage (Figure 3A,B). Similarly to the appearance, the SHOP treatments all effectively delayed the increase in the firmness and lignin content. After 50 d of storage, the firmness of the loquat was 13.99 N, 11.87 N, 11.52 N, and 11.03 N in the control, 90% SHOP, 70% SHOP, and 80% SHOP fruits, respectively. Meanwhile, the lignin content was 23.1%, 14.2%, and 10.5% lower in the 80% SHOP, 70% SHOP, and 90% SHOP fruits than in the control fruits at 50 d storage.

Generally, lignification is due to lignin accumulation in the cell wall [39]. The accumulation of lignin content leads to the destruction of the cell structure and the increase in firmness [41]. In the present study, the SHOP treatment could suppress the increase in firmness and effectively relieved the increase in lignin, maintaining a better appearance in the refrigerated loquat fruit, which was in accordance with the effect of methyl jasmonate, 1-methylcyclopropene, CaCl_2_ treatment, and glycine betaine treatment [42,43,44,45]. Thus, SHOP treatment may stimulate the resistance system to produce an effector response regulating lignin synthesis, which reduced the firmness and lignin accumulation and alleviated chilling injury induced by lignin accumulation.

### 3.3. High Lignin Synthesis Enzyme Activities

**Figure 4:**
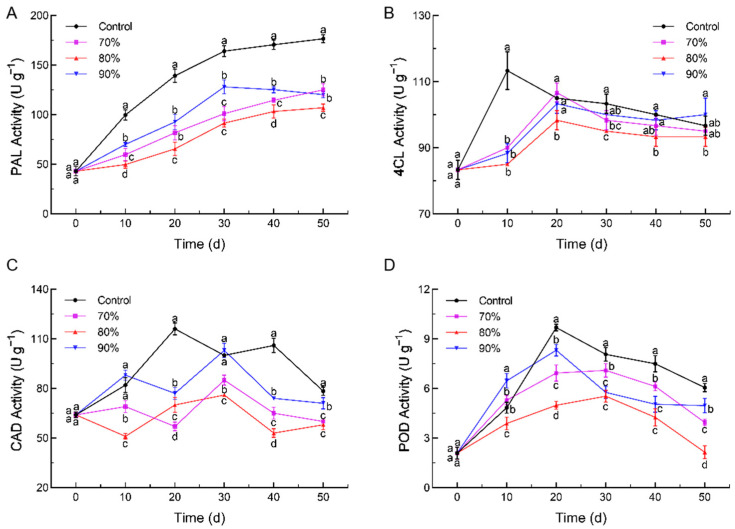
Effect of short-time high-oxygen pre-treatment (SHOP) on PAL activity (**A**), 4CL activity (**B**), CAD activity (**C**), and POD activity (**D**) of loquat fruit. The results are expressed as means ± standard deviation. Different letters represent significant differences among four groups (*p* ≤ 0.05).

The changes in the PAL, 4CL, CAD, and POD enzyme activities in lignin synthesis through the storage period are shown in Figure 4. The PAL activity of the loquat fruit followed a generally upward trend over the storage time (Figure 4A). The different levels of SHOP markedly restrained the rise in the PAL activity more than in the control. At the end of storage, the PAL activities in the 80%, 90%, and 70% SHOP fruits were 39.4%, 31.9%, and 29.2% lower than those in the control fruits. Meanwhile, SHOP reduced the activity of 4CL and delayed the 4CL peak during loquat storage until 20 d (Figure 4B). Among them, the 80% SHOP fruits had the lowest 4CL activity throughout storage. For the CAD and POD activities, their peaks were effectively retarded to 30 d in the 70% and 80% SHOP samples (Figure 4C,D). Notably, the POD activity in the 80% SHOP fruits was 64.6% less compared to that in the control fruits by the end of the storage. Moreover, the CAD and POD activities during the first ten days were higher in the 90% SHOP fruits than in the other pre-treatments, which could be due to high oxygen as an abiotic stress stimulating the cellular stress response.

These results revealed that the SHOP treatment regulated the enzyme activities of lignin synthesis enzymes. Lignin synthesis enzymes play different roles in regulating lignin. PAL plays a crucial role in the initial step of lignin biosynthesis, while 4CL is a key enzyme that connects the phenylpropanoid metabolic pathway to the specific lignin synthesis pathway. Additionally, CAD catalyzes the reduction of cinnamaldehydes to their corresponding alcohols, and POD is responsible for polymerizing monolignols into lignin macromolecules [46]. Previous research has demonstrated that lignification could be effectively delayed by inhibiting enzyme activities involved in the phenylpropanoid pathway [47,48]. The SHOP treatment inhibited the enzyme activities of the phenylpropane pathway, which also aligned with our theory. Notably, feedback regulation of the oxidant system resulted in the reduction in the enzyme activities of PAL and 4CL after the antioxidative system in loquat fruit was activated by short-time high oxygen [49]. Furthermore, the POD enzyme activity demonstrated a trend of first increasing and then decreasing, which was similar to the results of high-oxygen pre-treatment in fresh-cut potatoes [50]. Meanwhile, the POD activity in the 90% SHOP group was higher than in the other groups during early storage, indicating the higher sensitivity of POD to oxygen, and that high oxygen levels lead to the cellular stress response, which is consistent with the results of the microscopic structural observations [22]. Moreover, compared to the 70% and 90% SHOP treatments, the 80% SHOP treatment exhibited the most potent inhibition of the PAL, 4CL, CAD, and POD activities, suggesting that the optimal oxygen concentration is crucial for modulating lignin biosynthesis enzyme activities in loquat.

### 3.4. Effect of SHOP on Reactive Oxygen Species (ROS)-Related Enzyme Activities and MDA Content

**Figure 5:**
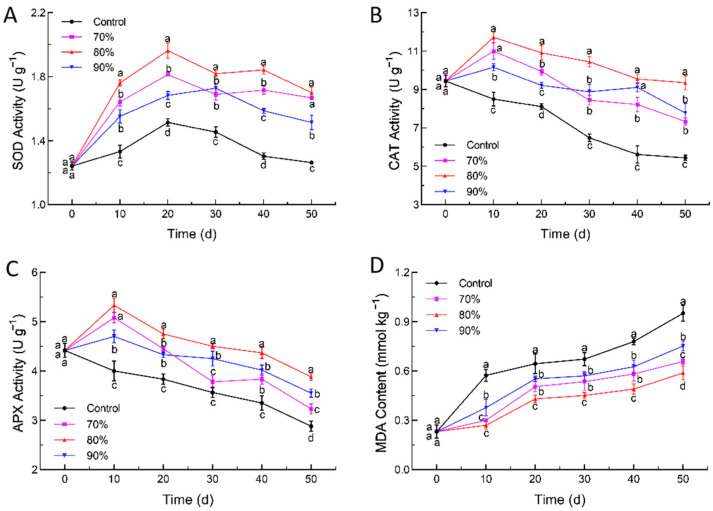
Effect of short-time high-oxygen pre-treatment (SHOP) on SOD activity (**A**), CAT activity (**B**), APX activity (**C**), and MDA content (**D**) of loquat fruit. Data are expressed as mean ± standard deviation. Different letters denote significant differences among four groups (*p* ≤ 0.05).

The regulation of reactive oxygen species (ROS) pathways in fruits and vegetables helps to maintain the postharvest quality by enhancing the activity of antioxidant enzymes, and this process eliminates ROS accumulation and delays the aging of the fruit [51]. The enzyme activity changes in SOD, CAT, and APX are shown in Figure 5. The SOD enzyme activity initially increased and then decreased. The peak activities of SOD in the treatment groups with 80% SHOP, 70% SHOP, and 90% SHOP, and the control group were 1.96 U·g^−1^, 1.81 U·g^−1^, 1.73 U·g^−1^, and 1.51 U·g^−1^, respectively. The groups treated with SHOP showed a slower downward trend than control (Figure 5A). Similarly to SOD, the enzyme activities of catalase (CAT) and ascorbate peroxidase (APX) showed higher activity in the first 10 days and a decrease after that. The 80% high-oxygen treatment group maintained elevated levels of (CAT) and (APX) enzyme activity during storage, which contributed to the reduction in cellular tissue damage caused by reactive oxygen species (Figure 5B,C).

Generally, MDA is among the products of membrane lipid peroxidation, and a higher MDA content represents more serious cell membrane damage [52]. The MDA content of loquat fruit continued to increase throughout the storage period, as shown in Figure 5D. Its accumulation was slowed down by SHOP, in which 80% SHOP maintained the lowest MDA content throughout storage. After 50 d of storage, the MDA contents in the 80% oxygen, 70% oxygen, 90% oxygen, and control groups were 2.55, 2.85, 3.26, and 4.12 times the initial values, respectively (Figure 5D).

In the current study, the SHOP treatment induced the activities of SOD, CAT, and APX compared to the control group while also delaying the accumulation of MDA. This indicated that high oxygen, as a resistance-inducing measure, activates a stress response mechanism that enhances these enzymatic activities. The enhancement of antioxidant enzyme activity reduces the levels of reactive oxygen species, thereby attenuating the signaling role of reactive oxygen species, which in turn maintains membrane integrity and inhibits lignin biosynthesis [27,53]. Meanwhile, due to the high oxygen stimulating the resistance system, the loquat fruit exhibited elevated free radical levels, which led to a substantial increase in the activities of the antioxidant enzyme system in the SHOP groups, such as those of APX, CAT, and SOD at the point of initial storage [54]. However, the weakening of the oxidative stress responses led to a decline in the enzyme activity with the increase in storage time, which showed the same trends as those in the control in the late storage [55]. Worthy of note, the treatment effect and oxygen concentration were not simply positively correlated. The 80% SHOP group consistently induced peak enzymatic responses and the enzyme activities were decreased at a concentration of 70% SHOP and 90% SHOP. In a relatively low-oxygen environment, the reduced oxidative stress leads to a decreased demand for antioxidant enzymes, resulting in a decline in their activity. Conversely, in a higher-oxygen environment, excessive reactive oxygen species (ROS) induce oxidative damage, which subsequently inhibits the function of antioxidant enzymes [56]. Maintaining an optimal oxygen concentration is crucial for delaying the senescence of loquat and preserving its quality.

### 3.5. Fruit Quality

**Figure 6:**
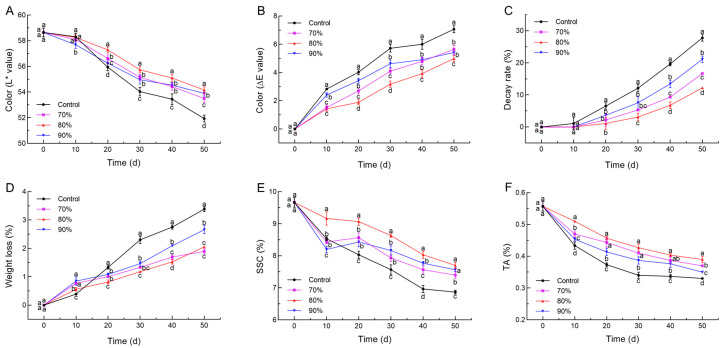
Effect of short-time high-oxygen pre-treatment (SHOP) on lightness change in color (L* value) (**A**), overall change in color (ΔE value) (**B**), decay rate (**C**), weight loss (**D**), SSC (**E**), and TA (**F**) of loquat fruit. Data are expressed as mean ± standard deviation. Different letters denote significant differences among four groups (*p* ≤ 0.05).

The effect of SHOP on the surface color of the loquat was investigated (Figure 6A,B). Consistent with the appearance observations, a continuous decrease in the L* values was observed, and the application of 80% SHOP maintained the highest L* during the storage (Figure 6A). For the color change, the ΔE* of the SHOP groups significantly decreased during the storage time, and the 80% SHOP had more effect on the ΔE* than the other treatments (Figure 6B).

As shown in Figure 6C, the decay rate showed an increasing trend during the entirety of storage in all the groups. At the end of storage, the decay rate of the loquat was 56%, 40.0%, and 24% lower in the 80%, 70%, and 90% SHOP groups than in the control group, respectively. The decay rate of the loquat receiving 80% SHOP treatment was significantly reduced compared to that of other groups. Meanwhile, after 50 days of storage, the weight loss of the loquat continued to increase, and the 80% SHOP fruits had the lowest level (Figure 6D).

SSC and TA are essential substances that constitute the loquat fruit’s taste and nutritional quality. SSC denotes soluble sugars, and TA mainly refers to its organic acids. As shown in Figure 6E, the SSC of the loquat fruit showed an overall decreasing trend. After storage for 20 d, SHOP maintained high SSC levels. TA gradually decreased in all the groups during storage (Figure 6F), with the loquat treated with SHOP maintaining higher TA, and the 80% SHOP treatment was the most effective in reducing SSC and TA depletion.

Overall, SHOP effectively maintained the loquat quality by preserving the color, reducing decay, and maintaining water and nutrients. The L* of the loquat decreased throughout storage, correlated with physiological alterations in postharvest ripening and senescence and the accumulation of carotenoids [57]. Additionally, at the end of storage, the lower weight loss played a positive role in maintaining the quality of loquat, which was related to the 80% SHOP treatment maintaining better cell integrity, and the intercellular spaces of the cells with better water retention had more water [22]. Notably, the inhibition of the decay rate in the loquat was consistent with results in fresh goji berries pre-treated with short-time high oxygen, showing that SHOP, as a form of stress, increases the resistance and affects the community composition of loquat fruit, thereby effectively inhibiting postharvest microbial contamination and inhibiting the decay rate [23].

## 4. Conclusions

In conclusion, the short-time high-oxygen pre-treatment at an optimal concentration (80%) was the most effective method of alleviating lignification and preserving the loquat quality over 50 days of storage at 4 °C. The 80% SHOP treatment effectively maintained the loquat appearance, quality, and cell structure while inhibiting lignin accumulation and firmness, which was increased by inhibiting the activities of key lignin synthesis enzymes (PAL, 4CL, CAD, and POD) and enhancing the activities of reactive oxygen species (ROS)-related enzymes (SOD, CAT, and APX). Meanwhile, 80% SHOP could reduce the MDA content, maintain the cell structure integrity, and improve the storage quality characteristics, such as the color, decay, weight loss, SSC, and TA. As a whole, the optimal concentration of short-time high-oxygen pre-treatment offers a simple, effective, safe, and cost-efficient method for delaying lignification and extending the shelf life of loquat.

## Data Availability

The original contributions presented in this study are included in the article. Further inquiries can be directed to the corresponding author.
